# Revealing Erythema Nodosum Leprosum in a Leprosy Patient: A Case of Treatment Noncompliance

**DOI:** 10.1155/crdi/1071013

**Published:** 2025-10-15

**Authors:** Bassem Alhariri, Memon Noor Illahi, Namaa Abubaker Suliman Elshaikh, Rashid Gamal Mohamed Omer, Noof AlQahtani, Muhammad Sharif

**Affiliations:** ^1^Department of Medicine, Hamad Medical Corporation, Doha, Qatar; ^2^College of Medicine, Qatar University, Doha, Qatar; ^3^College of Medicine, Weill Cornell Medicine–Qatar, Doha, Qatar

**Keywords:** Erythema Nodosum Leprosum, immune complex, lepromatous leprosy, leprosy, Type 2 Lepra reaction

## Abstract

Lepra reactions (LR) are acute inflammatory conditions with immune mediators that are highly morbid. Patients with the lepromatous end of the leprosy spectrum (BL-LL) are the only ones who develop Type-2 LR. 90% of the time, it happens during or right after treatment, usually within 2 years. The emergence of Erythema Nodosum Leprosum (ENL) suggests a paradoxical immune reaction and hypersensitivity to leprosy bacteria that results in painful erythematous nodules and systemic symptoms. Here, we present a case of Type 2 LR in a 41-year-old Indian man diagnosed with lepromatous leprosy 2 years ago. He was treated with MDT regime for 12 months and was compliant with treatment till 1 month before presenting with fever, cough, red eyes, and painful erythematous nodules involving his trunk and extremities. This case underscores the severe systemic inflammatory response that can be triggered by noncompliance with MDT, mimicking sepsis and requiring prompt recognition and management. Early recognition of ENL and appropriate management are essential to alleviate symptoms and prevent serious complications that may result from this serious condition.

## 1. Introduction

Leprosy, also known as Hansen's disease, is a chronic infectious disease caused by *Mycobacterium leprae* that primarily affects the skin, peripheral nervous system, upper respiratory tract, eyes, and testes [1]. According to the World Health Organization (WHO), more than 120 nations still have cases of this neglected tropical disease, with over 200,000 new cases reported annually [2]. In 2019, Brazil, India, and Indonesia reported over 10,000 new cases [3]. Lepra reactions (LR), which are acute inflammatory reactions that might complicate leprosy, are a major characteristic of the disease. The two main types of LR are Type 1 (reversal reaction) which typically occurs in tuberculoid (TL) and borderline tuberculoid leprosy (BT), and Type 2 also known as Erythema Nodosum Leprosum (ENL) [1, 4–6]. ENL is a delayed immune-complex-mediated response to high levels of circulating antimycobacterial antibodies, leading to systemic inflammation and multisystem involvement [5, 7]. Clinically, this multisystem disorder usually presents as erythematous, painful papules and nodules in the extremities of approximately 50% of individuals with lepromatous leprosy (LL) and, less frequently, in about 25% of those with borderline lepromatous leprosy (BL) [6]. Apart from skin nodules, patients may present with other systemic manifestations such as fever, iritis, arthritis, lymphadenitis, orchitis, and neuritis [1]. The diagnosis of ENL is mainly clinical, supported by the presence of leprosy and exclusion of other conditions. Despite the availability of effective treatments for leprosy, ENL has an important incidence and demands further understanding in terms of its pathophysiological and clinical presentation and therapeutic approaches.

## 2. Case Presentation

A 41-year-old Indian gentleman came in presenting with fever, cough, vomiting, eye redness, and generalized painful rash all over his body. Symptoms started 1 month ago. The patient was diagnosed with Hansen's disease LL (Type 2 LR) back in November 2022 during a visit in India. The diagnosis was based on clinical and histopathological findings. His baseline Bacteriological Index (BI) was reported to be 4+; however, records of the Morphological Index (MI) were not available. He was then started on the WHO multidrug treatment (MB-MDT) regimen for multibacillary leprosy (rifampicin, clofazimine, and dapsone) with a planned duration of 12 months; however, he stopped taking medications 1 month ago just before experiencing current symptoms. He has missed his scheduled follow-ups as he travelled back to his home country. The patient also complains of loss of appetite but denies any unintentional loss of weight. He has also been diagnosed with Type 2 diabetes mellitus, which is moderately controlled, and is on metformin. He is a nonsmoker and does not drink.

## 3. Physical Examination

According to the patient's vital signs on admission, the patient had borderline hypotension with systolic blood pressure of 99 mmHg. Other vitals were unremarkable.

**Figure 1:**
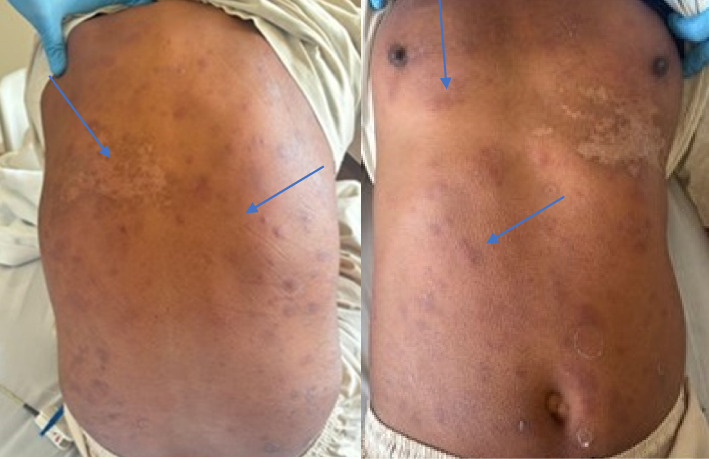
Showing multiple erythematous, raised, tender nodules all over his body, more notably on the back and abdomen (blue arrows).

On skin examination, there were multiple erythematous, raised, tender nodules all over his body and face, more notably on the back (Figure 1). Cardiovascular, respiratory, neurological, and abdominal examinations were insignificant (Table 1).

The clinical impression at the time of examination was sepsis versus an immune reaction. Significant laboratory findings include leucocytosis, elevated inflammatory markers, and hyponatremia (Table 2).

**Figure 2:**
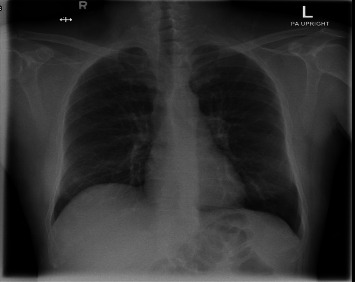
Chest X-ray: showing unremarkable chest X-ray on admission.

A chest X-ray and Echocardiography, which turned out unremarkable (Figure 2), were done on admission to rule out pneumonia and any cardiac pathology.

Blood samples were taken for blood culture which showed no growth.

### 3.1. In-Patient Management

Patients started on IV antibiotics (ceftriaxone and azithromycin) for empirical coverage of sepsis, steroids (intravenous methylprednisolone 40 mg every 8 h), insulin sliding scale, and IV fluids. Antibiotics were discontinued after 48 h once blood cultures were negative and the diagnosis of ENL was established.

Dermatology was consulted, and a biopsy was taken from the left upper back for pathology review. Later, the results of the biopsy showed unremarkable epidermis with dermal epithelioid histiocytes surrounding small cutaneous nerves, vessels, and adnexal structures. Macrophages are found in poorly circumscribed masses in the dermis with few lymphocytes. Slit-skin smears were not performed during this acute admission.

On the third day, the patient was discharged with referral to the infectious disease clinic for further follow up. Discharge plan was to continue oral prednisolone 60 mg daily with a taper plan of 10 mg weekly over 6 weeks taper off prednisolone at home. Patient was also advised to discontinue antibiotics as cultures were negative continue the oral antibiotics for 3 more days and continue with oral hypoglycaemic medication.

After 1 week, the patient was seen in the infectious disease clinic, where he was restarted on the WHO MB-MDT regimen for a full 12-month course alongside multidrug treatment regimen with thalidomide 100 mg twice daily for 2 more years as a steroid-sparing agent for long-term ENL control and stopped his current treatment regimen from India. Further follow-up was planned every other 4 weeks.

## 4. Discussion

Type 2 LR, referred to as ENL reaction, is an immune-complex-mediated inflammatory response that usually occurs in patients having LL with high load of leprosy bacilli. ENL may develop at any time of the disease course: before, during, or after initiation of treatment with MDT. According to the literature, 90% of cases acquired ENL after starting treatment, usually within 2 years [1, 4, 8]. The abrupt cessation of MDT, particularly rifampicin, likely led to a rapid change in the antigenic load and a surge in immune complex formation, precipitating this severe ENL reaction. The systemic inflammatory response in ENL, driven by a cytokine storm, can clinically mimic sepsis, as seen in this case with leukocytosis, markedly elevated CRP, and fever, explaining the initial diagnostic challenge.

The patient's comorbid type 2 diabetes mellitus may have contributed to an altered immune state, potentially facilitating both the initial infection and the severity of the inflammatory reaction upon treatment interruption.

When it comes to its pathophysiology, ENL appears to be a complex interaction between various aspects of the immune system. For the past years, theories have considered the role of neutrophils and immune-complex formation as a hallmark of ENL. Recent studies showed a multitude of factors such as proinflammatory cytokines, Treg cells, TLR-9, CCL-5, IFN-γ, and even possibly B cells, bringing humoral immunity into the picture [6, 9, 10]. Furthermore, genetic predisposition and environmental factors may influence the likelihood of developing ENL, suggesting that the pathophysiology is multifactorial and is still unclear [11].

Clinically, ENL presents as crops of multiple erythematous to dark brown, painful papules and nodules over the face commonly and predominantly on the extensor surface of the limbs [4]. Fever, malaise, arthritis, neuritis, vasculitis, uveitis, lymphadenopathy, and SIRS are other systemic signs of ENL [12]. Our patient was diagnosed with LL 2 years prior and ceased his treatment 1 month before presenting with this painful erythematous nodule involving his trunk and limbs, which is likely triggered by fluctuations in immune response following discontinuation of treatment. These nodules were preceded by fever, cough, and eye redness. This wide range of clinical presentations highlights the complex nature of ENL and the significance of maintaining a high level of suspicion of ENL in patients with LL. Patients with ENL may experience recurrent outbreaks that correspond with changes in their immunological status, often necessitating adjustments in treatment.

Regarding management of ENL, it is crucial to initiate appropriate anti-inflammatory treatment while addressing the underlying leprosy. The WHO's guidelines for treatment of ENL-complicated LL involve corticosteroids as the first-line treatment as they are effective in alleviating symptoms, reducing inflammation and preventing irreversible nerve damage [10, 13]; however, their use must be carefully monitored due to the potential fluctuations in immune function which can results in emergence of new nodules and other complications. Thalidomide has a considerable efficacy in men and women of nonreproductive potential; however, its use is limited due to its teratogenesis [9, 13]. In addition to these treatments, it is essential to re-initiate MDT for LL, as stopping treatment can lead to the progression of the disease and further complications. Our patient was on MDT till 1 month before the appearance of skin nodules. He was started on high dose of prednisolone and after 1 week, he was seen in the outpatient clinic, and all his lesions had subsided. He was restarted on MDT with thalidomide, and his steroid therapy was tapered. This rapid improvement in the patient symptoms highlighted the vital role of patient education, treatment adherence and regular follow up in enhancing the patient outcomes.

In this case, high-dose corticosteroids were initiated first to rapidly control the severe inflammation. Thalidomide was subsequently chosen for long-term ENL management due to its proven efficacy, its role as a steroid-sparing agent, and its suitability for this male patient, thus avoiding the risks associated with long-term high-dose steroid therapy. The decision to restart a full 12-month course of WHO MB-MDT was imperative to treat the underlying multibacillary disease, as the patient had not completed the standard regimen and was at high risk for relapse. Drug resistance testing was not performed due to lack of clinical suspicion (no prior MDT failure) and limited availability.

This case highlights that treatment nonadherence is a critical risk factor for severe ENL reactions. It underscores the necessity of patient education, structured follow-up programs, and a multidisciplinary approach to ensure compliance and prevent serious complications.

## 5. Conclusion

ENL remains a complex and critical complication of LL, reflecting the intricate relationship between the immune system and persistent infection. This case illustrates the importance of recognizing various systemic manifestations of ENL in patients with LL. A thorough grasp of the pathophysiology, clinical implications, and management strategies is crucial for enhancing the quality of life for patients with this complex condition. Additionally, the multidisciplinary approach, involving dermatologists, infectious disease specialists, and possibly ophthalmologists, is crucial in providing comprehensive care for this patient.

## Figures and Tables

**Table 1:** Physical examination on admission.

General examination	Conscious, alert, oriented, and appears well
Neurological examination	GCS 15/15; no focal neurological deficits
Cardiovascular examination	Normal S1 and S2 with no added sounds
Respiratory examination	Air entry is present bilaterally with no added sounds
Abdominal examination	Soft, nontender with no guarding or rebound tenderness
Skin examination	Burning erythematous raised annular skin rash with no signs of ulnar, fibular or greater auricular thickening note. No mouth ulcers noted

**Table 2:** The results of laboratory investigations on admission.

Test	Result (units)	Reference range	Flag
WBC (white blood cell count)	16.4 × 10^3^/uL	4.0–11.0	High
RBC (red blood cell count)	3.6 × 10^6^/uL	4.5–5.9	Low
Hgb (hemoglobin)	9.7 g/dL	13.5–17.5	Low
Hct (hematocrit)	30.3%	40–50	Low
MCV (mean corpuscular volume)	84.9 fL	80–100	Normal
MCH (mean corpuscular hemoglobin)	27.2 pg	26–34	Normal
MCHC (mean corpuscular hemoglobin concentration)	32 g/dL	32–36	Normal
RDW-CV (red cell distribution width)	17.7%	11.5–14.5	High
Platelet	232 × 10^3^/uL	150–400	Normal
MPV (mean platelet volume)	12 fL	7.5–11.5	High
PDW (platelet distribution width)	15.6 fL	9.0–14.0	High
ANC (absolute neutrophil count)	16 × 10^3^/uL	1.5–8.0	High
Lymphocyte count	0.2 × 10^3^/uL	1.0–4.0	Low
Monocyte count	0.1 × 10^3^/uL	0.2–1.0	Low
Eosinophil count	0.01 × 10^3^/uL	0.0–0.5	Low
Basophil count	0.03 × 10^3^/uL	0.0–0.1	Normal
Neutrophil %	97.5%	40–75	High
Lymphocyte %	1.5%	20–40	Low
Monocyte %	0.7%	2–8	Low
Eosinophil %	0.1%	0–6	Normal
Basophil %	0.2%	0–2	Normal
Prothrombin time (PT)	16.8 s	10–14	High
INR (international normalized ratio)	1.5	0.9–1.1	Normal
APTT (activated partial thromboplastin time)	36.7 s	25–35	High
Urea	5.2 mmol/L	2.5–7.5	Normal
Creatinine	78 umol/L	60–110	Normal
Sodium	128 mmol/L	135–145	Low
Potassium	4.2 mmol/L	3.5–5.0	Normal
Chloride	99 mmol/L	95–105	Normal
Bicarbonate	13 mmol/L	22–30	Critical
Calcium	2.14 mmol/L	2.1–2.6	Normal
Adjusted calcium	2.46 mmol/L	2.1–2.6	Normal
Bilirubin total (T)	35 umol/L	5–21	High
Bilirubin direct (D)	20 umol/L	0–6	High
Total protein	67 g/L	60–80	Normal
Albumin	24 g/L	35–50	Low
Alkaline phosphatase (Alk Phos)	107 U/L	45–115	Normal
ALT (alanine aminotransferase)	24 U/L	7–56	Normal
AST (aspartate aminotransferase)	26 U/L	10–40	Normal
B-Hydroxybutyrate	2.53 mmol/L	0.02–0.27	High
Glucose	6 mmol/L	3.9–6.1	High
HbA1c	5.2%	4.0–5.6	Normal
CRP (C-reactive protein)	342.2 mg/L	0–10	High
Lactic acid	1.1 mmol/L	0.5–2.2	Normal
Procalcitonin	1.46 ng/mL	< 0.05	High

## Data Availability

The data that support the findings of this study are available in this article.
